# Impact of moral case deliberation in healthcare settings: a literature review

**DOI:** 10.1186/s12910-018-0325-y

**Published:** 2018-11-06

**Authors:** Maaike M. Haan, Jelle L. P. van Gurp, Simone M. Naber, A. Stef Groenewoud

**Affiliations:** Radboud university medical center, Radboud Institute for Health Sciences, IQ healthcare, Geert Grooteplein 21, P.O. Box 9101 (114), 6500 HB, Nijmegen, The Netherlands

**Keywords:** Health personnel (MeSH), Caregivers (MeSH), Healthcare professionals, Clinical ethics (MeSH), Clinical ethics support, Moral case deliberation, Outcomes, Moral reflection

## Abstract

**Background:**

An important and supposedly impactful form of clinical ethics support is moral case deliberation (MCD). Empirical evidence, however, is limited with regard to its actual impact. With this literature review, we aim to investigate the empirical evidence of MCD, thereby a) informing the practice, and b) providing a focus for further research on and development of MCD in healthcare settings.

**Methods:**

A systematic literature search was conducted in the electronic databases PubMed, CINAHL and Web of Science (June 2016). Both the data collection and the qualitative data analysis followed a stepwise approach, including continuous peer review and careful documentation of our decisions. The qualitative analysis was supported by ATLAS.ti.

**Results:**

Based on a qualitative analysis of 25 empirical papers, we identified four clusters of themes: 1) facilitators and barriers in the preparation and context of MCD, i.e., a safe and open atmosphere created by a facilitator, a concrete case, commitment of participants, a focus on the moral dimension, and a supportive organization; 2) changes that are brought about on a personal and inter-professional level, with regard to professional’s feelings of relief, relatedness and confidence; understanding of the perspectives of colleagues, one’s own perspective and the moral issue at stake; and awareness of the moral dimension of one’s work and awareness of the importance of reflection; 3) changes that are brought about in caring for patients and families; and 4) changes that are brought about on an organizational level.

**Conclusions:**

This review shows that MCD brings about changes in practice, mostly for the professional in inter-professional interactions. Most reported changes are considered positive, although challenges, frustrations and absence of change were also reported. Empirical evidence of a concrete impact on the quality of patient care is limited and is mostly based on self-reports. With patient-focused and methodologically sound qualitative research, the practice and the value of MCD in healthcare settings can be better understood, thus making a stronger case for this kind of ethics support.

## Background

In healthcare, professionals are frequently confronted with morally complex and sometimes tragic situations in which difficult treatment and care decisions with far-reaching consequences have to be made [1]. Clinical ethics support (CES) helps in dealing with these complex issues. Over the past years, interest in CES has increased worldwide [2]. CES currently has many forms. A useful distinction was made by Rasoal et al. [2], who distinguished between ethics support services using a top-down and a bottom-up approach. Examples of a *top-down approach* are clinical ethics consultations and ethics committees, more common in the United States. In this top-down approach, according to Rasoal et al., the involved ethicist is generally attributed an expert position and advises professionals, although the actual expertise in CEC and the exact role of the ethicist is debated [2]. The outcomes of these consultations are to benefit patients and families, whereas healthcare professionals profit only indirectly from participating [3]. In contrast, group deliberations (such as moral case deliberation, ethics rounds, reflections or discussion groups) are an example of ethics support services with a *bottom-up approach*. These services have been reported mostly from European communities. Here, the ethicist facilitates the conversation without having an advisory role. The focus is on the reflection process of healthcare professionals, more than on a decision or solution for a clinical problem [3].

Moral case deliberation fits the latter approach. MCD is a collaborative meeting where a group of healthcare professionals jointly reflects on a concrete moral question, issue or dilemma. Essentially, and in contrast to other kinds of (more informal) meetings, a moral case deliberation is structured by a conversation method and moderated by a facilitator, often an ethicist [4–9]. For a recent case example of how a specific conversation method of MCD works in practice, we refer to Tan et al. [10]. During such a deliberation, as well as during similarly organized group sessions, professionals have the opportunity to freely articulate and share their stories, experiences, opinions and perspectives [9, 11–17]. For the remainder of this paper, we will use the term moral case deliberation (from here-on referred to as “MCD”) as an umbrella term for all variations of group deliberations with a specific focus on moral issues in healthcare.

Silén et al. [18] point to the importance of evaluating CES services and question whether it is defensible to conduct group deliberations that are time-consuming, without some form of proof of value for the healthcare practice. In recent years, research has been conducted on evaluating group deliberations in terms of quality of conversation [19–21]. However, thorough empirical evidence with regard to the impact of MCD seems limited [18, 22]. For the existing practice of MCD in healthcare organizations, it is necessary to substantiate its value, partly grounded in empirical evidence.

This literature review was conducted to gain insight into what has already been investigated in previous studies of the impact of MCD. The research question central to this review is the following: what is the impact of moral case deliberation with groups of healthcare professionals in a clinical setting? With this literature review, we aim to investigate the empirical evidence of MCD, thereby a) informing the practice, and b) providing a focus for further research on and development of MCD in healthcare settings.

## Methods

### Design

**Fig. 1:**
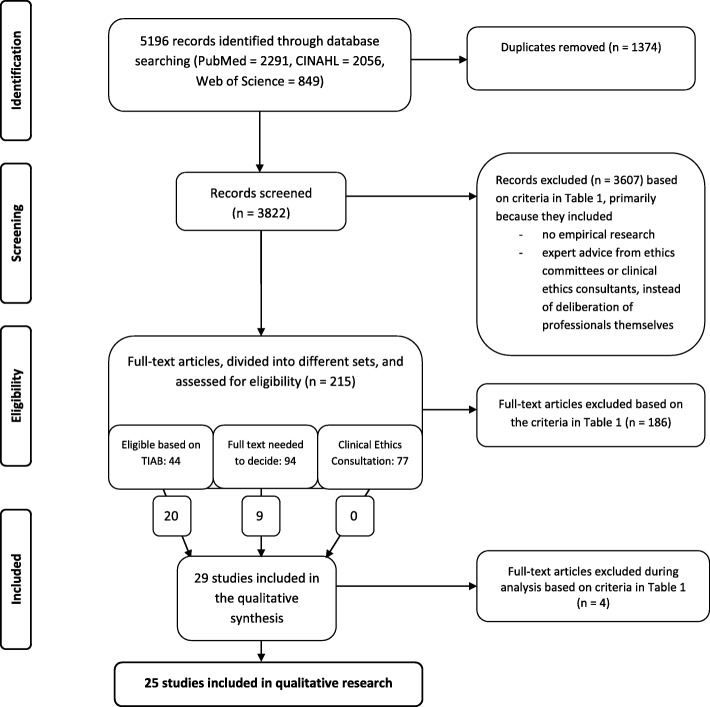
Preferred Reporting Items for Systematic Reviews and Meta-Analyses (PRISMA) flow diagram

This review’s research question focuses on the impact of moral case deliberation by groups of healthcare professionals. Here, we define impact as the changes that are brought about by participating in MCD. Since changes can be operationalized in several ways, we chose to integrate both quantitative and qualitative papers, based on the integrative review approach by Whittemore and Knafl [23]. We adopted a systematic, stepwise approach, including continuous peer review and careful documentation of our decisions in order to comply with the prescribed analytic honesty, e.g., making the thoughtful analysis process transparent [23]. A PRISMA flow chart of the research process can be found in Fig. 1. The review was registered in the PROSPERO database (CRD42016043531), an international prospective registry of systematic reviews, in July 2016.

### Literature search

A literature search was conducted in the electronic databases PubMed, CINAHL and Web of Science (All Databases) in June 2016 (see Table 1). As ‘moral case deliberation’ as a description is used broadly, and possibly similar forms of ethics support services may be described with different terminologies [24], several equivalent terms for moral deliberation within groups of healthcare professionals were piloted and then used in the search. We deliberately included ‘clinical ethics consultation’ in the search string. This was to enable ourselves to explore whether moral case deliberation is conducted within the Anglo-Saxon clinical ethics consultation practice and whether or not it is justified to strictly separate this practice from the MCD-practice. Furthermore, in PubMed and CINAHL, the search was narrowed with synonyms and other words relating to the ‘impact’ of MCD. Locating as much research on our topic as possible is in line with Hawker et al.’s view of a literature search, thus reducing the amount of missed relevant insights because of vague descriptions or differences in terminology [25]. All search queries were limited to publications in English, German or Dutch. No date restrictions were used.

**Table 1:** Search strategy in databases

Basic search strategy in all three databases moral deliberation*OR moral case deliberation* OR ethical deliberation* OR ethical case deliberation* OR ethics deliberation* OR ethics case deliberation* OR ethical round* OR ethics round* OR ethical case discussion* OR moral case discussion* OR moral reflection* OR ethical reflection* OR ethics reflection* OR ethical decision making OR ethical case decision making OR ethical decisionmaking OR ethical case decisionmaking OR moral decision making OR moral case decision making OR moral decisionmaking OR moral case decisionmaking OR ethics meeting* OR ethics support OR clinical ethics support OR ethical support OR ethical reasoning OR ethical dialogue* OR ethical case review* OR ethical conversation* OR ethics conversation* OR moral conversation* OR ethics consultation [Mesh] *(in PubMed)* OR ethics consult* *(in CINAHL and Web of Science)* *Note: these terms were added with TIAB (PubMed), TI or AB (CINAHL) and TS (Web of Science).*	
AND-addition in PubMed and CINAHL (added with TIAB/TI/AB)	AND-addition in Web of Science (added with TS)
theory OR practice* OR evaluat* OR method* OR contribut* OR evidence OR report* OR harvest OR outcome* OR impact OR effect* OR result* OR influence OR benefit OR significance OR use OR appreciation OR value* OR support OR goal* OR purpose* OR intention* OR motive* OR reason* OR meaning* OR relevance OR importance OR need* OR aim* OR learn* OR model* OR debate* OR technique* Note: In PubMed and CINAHL the terms ‘result*’ and ‘method’ were added only with TI because these terms are commonly used in abstracts.	physician* OR doctor* OR nurse* OR healthcare personnel OR healthcare provider* OR healthcare professional* OR health personnel OR team* OR staff* *Note: Because of the number of articles found, the search in Web of Science was not narrowed down with terms relating to impact. The basis search was only expanded with terms relating to healthcare since this database is not specifically related to the field of healthcare (as are PubMed and CINAHL).*

The asterisk (*) was used to retrieve variations of the term that start with the same letters. The symbol represents any group of characters (including no character)

### Data extraction

After duplicate removal, the retrieved records were screened for relevance. Preceding the formal screening against eligibility criteria, all authors evaluated a random sample of 100 titles and abstracts of the retrieved records in order to refine the criteria for inclusion and exclusion and to test for usefulness (see Table 2). Since our research question asks for a reported impact of MCD, we focused on empirical evidence only and excluded papers if the impact was only (theoretically) assumed. The retrieved records were not assessed on their (methodological) quality. In the first screening step, the retrieved records were divided among two researcher duos. Each duo independently screened the records for relevance based on title and abstract. To prevent bias, the author(s) and publication year were not known by the screeners. This step resulted in several sets of records to keep the screening process manageable (see Fig. 1). Records about clinical ethics consultation were kept in a specific set to verify our assumption about the clinical ethics consultation practice being markedly different from the MCD practice. In the second screening step, the full texts of selected records were screened by the first author (MH) and a second screener, again discussed in research duos and – in case of doubt – were discussed by all authors until consensus was reached.

**Table 2:** Criteria for inclusion and exclusion

1. Research papers in English, German or Dutch 2. Moral case deliberation, which we understand as: a. a shared professional deliberation or meeting between healthcare professionals (i.e., physicians, nurses and other (para)medic personnel) *Exclusion: individual reflections instead of deliberation in a group, deliberation only between ethicists or in ethics committees, or deliberation specifically focused on parents or relatives* b. about an ethical/moral dilemma or question *Exclusion: strictly legal or medical technical deliberations, scientific studies or general deliberations about ethical dilemmas* c. in a clinical setting (i.e., hospital or mental healthcare facility) concerning a specific case in the care of patients *Exclusion: research and animal care; education or training, except for professionals working in education or training directly related to moral or ethical reflection in a clinical setting* 3. Peer-reviewed studies *Exclusion: editorials and other texts that were not peer reviewed* 4. Empirical studies with statements about the impact (i.e., effect, evaluation, importance, meaning, value, et cetera) of that (method of) deliberation	

### Data analysis

To do justice to the variety of methods used in the retrieved papers and the various forms of impact reported, we applied a stepwise qualitative analysis using the Computer Assisted Qualitative Data Analysis Software ATLAS.ti. The first paper was coded in an open way by all authors. An initial codebook was developed based on this open coding (MH). The subsequent 15 papers were coded by dividing them into different rounds over three different researcher duos, with MH being part of every duo. During this process, the codebook was continuously developed and adapted. The remaining nine papers were coded by MH and checked by the other authors on a category-level. In case of a difference of opinion, discussion continued until a consensus was reached. Lastly, the authors formed themes based on the codebook, which were related to each other and then clustered. In the development of this clustering, the themes were further refined.

## Results

### Characteristics of the included studies and group conversations

Initially, 5196 records were retrieved. After duplicate removal, 3822 studies remained for screening of the title and abstract (see Fig. 1). After a thorough screening, and sometimes full-text reading, no papers included in the set of studies about clinical ethics consultation were included in the qualitative data analysis, mainly because the studies concerned expert advice by consultants instead of a deliberation among healthcare professionals. The stepwise screening process led to a final inclusion of 25 empirical papers, twenty-one from Europe and four from the US (see Table 3 for study characteristics). The included studies used both quantitative and qualitative research methods, including surveys, focus groups, interviews, observational studies, content analysis of conversation protocols, audio/visual tape recordings of group deliberations, or a mixed-methods design. Participants in these conversations often discussed a patient case in which an ethical issue arose, for example, when to withdraw treatment of a very ill patient or how to address an aggressive or noncompliant client. Conversations also concerned other kinds of dilemmas in patient care, such as communication issues between staff and nurses. The included studies contained both prospective and retrospective case discussions. Most evaluative studies were based on self-reports of participants of group deliberations, sometimes with a baseline and intervention design.

**Table 3:** Characteristics of the included studies

Reference number	Authors	Journal	Title	Country	Setting and participants of the conversation	Data collection and respondents	Conversation method	Type of case
28	Appelbaum & Reiser (1981)	Hospital & Community Psychiatry	Ethics rounds: a model for teaching ethics in the psychiatric setting	USA	Mental healthcare, multidisciplinary	Quantitative: questionnaires among participants	Presentation and discussion, no particular method mentioned	Patient cases, retrospective (occasionally active)
30	Baumann-Hölzle, Maffezzoni & Bucher (2005)	Acta Pædiatrica	A framework for ethical decision making in neonatal intensive care	Switzerland	Hospital care (neonatal IC), multidisciplinary	Quantitative: questionnaires among participants; survival comparison with controls	Locally developed framework for structured decision making	Patient cases, prospective
16	Bernthal, Russell & Draper (2014)	Journal of the Royal Army Medical Corps	A qualitative study of the use of the four quadrant approach to assist ethical decision-making during deployment	United Kingdom	Military hospital care, health professionals	Qualitative: analysis of case conference forms; interviews with participants	Four Quadrant Approach (4QA)	Patient cases
33	Dauwerse, Weidema, Abma, Molewijk & Widdershoven (2014)	HEC Forum	Implicit and explicit clinical ethics support in The Netherlands: a mixed methods overview study	The Netherlands, Norway	Various settings, multidisciplinary	Quantitative: surveys. Qualitative: interviews and focus groups with management and ethics support staff	No particular method mentioned	Not specified
27	De Boer, Van Blijderveen, Van Dijk, Duivenvoorden & Williams (2012)	Journal of Medical Ethics	Implementing structured, multiprofessional medical ethical decision-making in a neonatal intensive care unit	The Netherlands	Hospital care (neonatal IC), multidisciplinary	Quantitative: questionnaires with staff	Five-step procedure based on Utrecht model and Nijmegen method	Patient cases, prospective (fictional cases when no actual case was at hand)
12	Grönlund, Dahlqvist, Zingmark, Sandlund & Söderberg (2016)	HEC Forum	Managing Ethical Difficulties in Healthcare: Communicating in Inter-professional Clinical Ethics Support Sessions	Sweden	“Highly specialized healthcare clinic”, multidisciplinary	Qualitative: content-analysis of audio and video recorded ethics sessions	Several steps, no particular method mentioned	Patient cases
7	Janssens, Zadelhoff, Van Loo, Widdershoven & Molewijk (2016)	Nursing Ethics	Evaluation and perceived results of moral case deliberation: A mixed methods study	The Netherlands, Norway	Elderly care, multidisciplinary	Quantitative and qualitative: questionnaires with participants; interviews; focus groups	Dilemma method or Socratic dialogue	Patient cases
13	Jehle & Jurchah (2014)	Topics in Stroke Rehabilitation	Patient With a Devastating Embolic Stroke: Using Weekly Multidisciplinary Ethics Rounds in the Neuroscience Intensive Care Unit to Facilitate Care and Communication	USA	Hospital care (neuroscience IC), multidisciplinary	Quantitative: surveys, not specified	“FESOR” format: Facts, Ethical question, Stakeholders, Options, Re-evaluation	Patient cases
31	Levine, Scott & Curran (1977)	Pediatrics	Ethics rounds in a Children’s Medical Center: evaluation of a hospital-based program for continuing education in medical ethics	USA	Children’s hospital care, multidisciplinary	Quantitative and qualitative: content-analysis of discussions; interviews and surveys among participants	Presentation and discussion, no particular method mentioned	Patient cases, retrospective
32	Lillemoen & Pedersen (2015)	BMC Medical Ethics	Ethics reflection groups in community health services: an evaluation study	Norway	Community health services, not clearly specified	Qualitative: focus groups with 1) participants, 2) ethics facilitators and 3) service managers; content analysis of documentation and observations	Centre for Medical Ethics method	Patient cases; constructed cases
36	Maffezzoni, Wunder, Baumann-Hölzle & Stoll (2003)	Zeitschrift für Arbeits- und Organisations-psychologie	Gruppenprozesse bei Entscheidungen zur Levensfähigkeit von Neugeborenen – Eine formative Evaluation	Switzerland	Hospital care (neonatal IC), multidisciplinary	Quantitative and qualitative: questionnaires among participants; analysis of video recorded sessions and conversation protocols	Locally developed ethical decision-making model	Patient cases, prospective
34	Meyer-Zehnder, Barandun Schäfer, Albisser Schleger, Reiter-Theil & Pargger (2014)	Der Anaesthesist	Ethische Fallbesprechungen auf der Intensivstation Vom Versuch zur Routine	Switzerland	Hospital care (IC), multidisciplinary	Quantitative: questionnaires among participants; summary of conversation protocols	“METAP” format: Module, Ethik, Therapieentscheidung, Allokation, Prozess	Patient cases, prospective
4	Molewijk, Verkerk, Milius & Widdershoven (2008)	Medicine, Health Care and Philosophy	Implementing moral case deliberation in a psychiatric hospital: process and outcome	The Netherlands	Mental healthcare, multidisciplinary	Quantitative and qualitative responsive evaluation: a questionnaire among participants; interviews with various stakeholders; participant observation; document analysis	Structured conversation, no particular method mentioned	Patient cases, retrospective and prospective
5	Molewijk, Abma, Stolper & Widdershoven (2008)	Journal of Medical Ethics	Teaching ethics in the clinic. The theory and practice of moral case deliberation	The Netherlands	Mental healthcare	Responsive evaluation approach	Depending on the case	Patient cases, professional-centered issues, and organization-centered questions
18	Silén, Haglund, Hansson & Ramklint (2015)	Nordic Journal of Psychiatry	Ethics rounds do not improve the handling of ethical issues by psychiatric staff	Sweden	Mental healthcare, multidisciplinary	Quasi-experimental: questionnaires in both intervention and control groups	Approach of imaginative ethics, no particular method mentioned	Patient case or theme
14	Silén, Ramklint, Hansson & Haglund (2016)	Nursing Ethics	Ethics rounds: An appreciated form of ethics support	Sweden	Mental healthcare, multidisciplinary	Qualitative exploratory and descriptive design: interviews with participants	Approach of imaginative ethics, no particular method mentioned	Patient case or theme
17	Söderhamn, Kjøstvedt & Slettebø (2014)	Nursing Ethics	Evaluation of ethical reflections in community healthcare: A mixed-methods study	Norway	Community health services	Qualitative and quantitative: focus groups with participants; questionnaires with employees	Traffic light method and fishbowl method	Ethical dilemmas
35	Svantesson, Anderzén-Carlsson, Thorsén, Kallenberg & Ahlström (2008)	Journal of Medical Ethics	Interprofessional ethics rounds concerning dialysis patients: staff’s ethical reflections before and after rounds	Sweden	Hospital care (nephrology), multidisciplinary	Quantitative and qualitative: questionnaires; content analysis of open-ended questions	Ethics rounds, no particular method mentioned	Patient cases
29	Svantesson, Löfmark, Thorsén, Kallenberg & Ahlström (2008)	Journal of Medical Ethics	Learning a way through ethical problems: Swedish nurses’ and doctors’ experiences from one model of ethics rounds	Sweden	Hospital care (nephrology), multidisciplinary	Qualitative: interviews with participants	Ethics rounds, no particular method mentioned	Patient cases
15	Tanner, Albisser Schleger, Meyer-Zehnder, Schnurrer, Reiter-Theil & Pargger (2014)	Medizinische Klinik – Intensiv-medizin und Notfall-medizin	Klinische Alltagsethik – Unterstützung im Umgang mit moralischem Disstress? Evaluation eines ethischen Entscheidungsfindungsmodellsfür interprofessionelle klinische Teams	Switzerland	Hospital care (IC and geriatrics), multidisciplinary	Qualitative and quantitative: individual and group interviews with employees; questionnaires among employees	“METAP” format: Module, Ethik, Therapieentscheidung, Allokation, Prozess	Patient cases
8	Van der Dam, Abma, Molewijk, Kardol, Schols & Widdershoven (2011)	Nursing Ethics	Organizing moral case deliberation Experiences in two Dutch nursing homes	The Netherlands	Elderly care, multidisciplinary	Qualitative: interviews with managers; observations and (tape) recordings of sessions; focus groups with participants	Depending on the case	Patient cases
9	Van der Dam, Schols, Kardol, Molewijk, Widdershoven & Abma (2013)	Social Science & Medicine	The discovery of deliberation. From ambiguity to appreciation through the learning process of doing Moral Case Deliberation in Dutch elderly care	The Netherlands	Elderly care, multidisciplinary	Qualitative: interviews with managers and other stakeholders; observations and (tape) recordings of sessions; focus groups with participants	Depending on the case	Patient cases
26	Voskes, Evenblij, Noorthoorn, Porz & Widdershoven (2014)	Psychiatrische Praxis	Ethische Fall-Deliberation zu Freiheitseinschränkungen in der Psychiatrie. Dilemmata, Nutzen und Implementierung	The Netherlands, Switzerland	Mental healthcare, multidisciplinary	Qualitative: interviews and focus groups with participants/employees; observation of sessions	Dilemma method	Patient cases, retrospective and prospective
6	Weidema, Molewijk, Kamsteeg & Widdershoven (2013)	Nursing Ethics	Aims and harvest of moral case deliberation	The Netherlands	Mental healthcare, mono-disciplinary (nurses)	Qualitative and quantitative: interviews with managers; questionnaires with participants	Structured conversation, no particular method mentioned	Concrete work-based case
11	Wocial, Hancock, Bledsoe, Chamness & Helft (2010)	JONA’S Healthcare Law, Ethics, and Regulation	An evaluation of unit-based ethics conversations	USA	Hospital care (different nursing units), mono-disciplinary (nurses)	Quantitative and qualitative: a survey among employees; focus group with participants	Structured story-telling, no particular method mentioned	Ethically challenging situations in care

### Clusters of themes

**Fig. 2:**
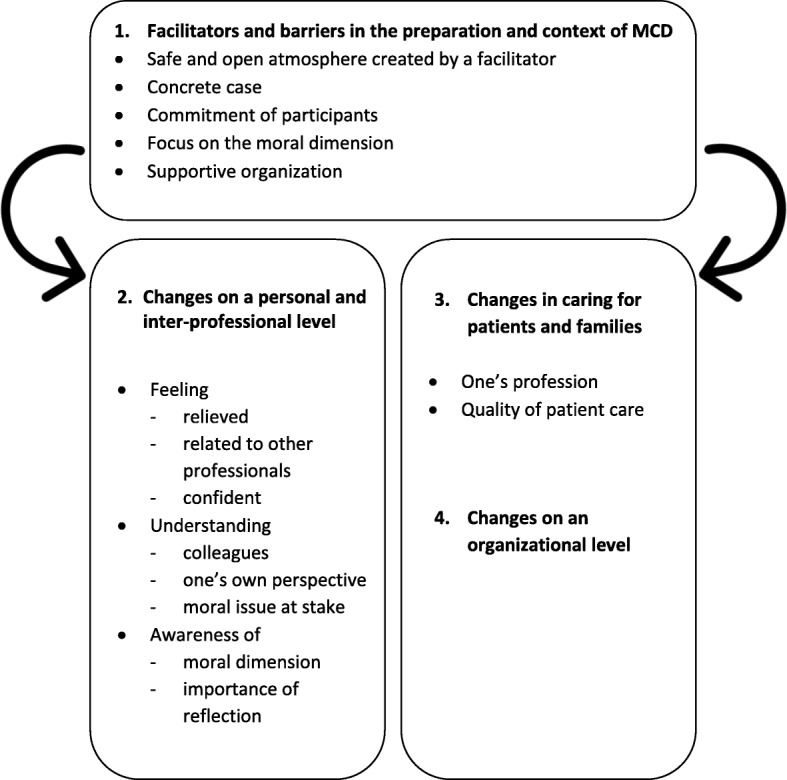
Clusters of the impacts of MCD

Our findings are divided into four clusters of themes related to impact, which are represented in Fig. 2:

1. Facilitators and barriers in the preparation and context of MCD
2. Changes that are brought about on a personal and inter-professional level
3. Changes that are brought about in caring for patients and families
4. Changes that are brought about on an organizational level

### Facilitators and barriers in the preparation and context of MCD

#### A safe and open atmosphere created by a facilitator

A moral case deliberation should be guided by a facilitator who is neutral with regard to the issue that is being discussed and who is not involved with or part of the team, to guarantee an atmosphere of trust [14, 26, 27]. Mutual trust may take some time, especially when participants stem from different disciplines and work in different wards [9]. It is deemed important that every participant gets the opportunity to speak out, without others feeling threatened or accused [14, 28].

#### A concrete case

The case to be discussed has to be concrete to allow participants to relate. Deliberations with only little reference to daily practice are usually disappointing for participants and are sometimes considered a waste of time [29]. In the study by Appelbaum et al. in 1981 [28], ongoing patient cases were explicitly not discussed to stimulate the participants’ ability to think abstractly. Nowadays, however, it is considered important to connect deliberations to daily practice in the wards, thus stimulating professionals to not revert ‘back to business as usual’ after the deliberation [8].

#### Commitment of participants

For an MCD to be successful it is important for the participating healthcare professionals to be committed and cooperative [14]. Discontinuity in attendance and absence of team members is seen as a barrier [7, 8, 13], preventing implementation of what is discussed or decided [8]. Adequate preparation and information promotes involvement in the discussion [7]. The interdisciplinary character of such deliberations is often experienced as positive [15, 26, 30, 31]. However, this may also hinder discussion because of differences of opinion regarding ethical, legal, social or medical aspects [31].

#### A focus on the moral dimension

In contrast to, for example, practical, legal, economical or psychological issues, a moral issue concerns the question: “What is a ‘good’ thing to do in this particular case/situation?”. Should we consider discontinuation of life-sustaining treatment for this patient? What does it mean to provide ‘good care’ to this aggressive client? Is it appropriate to treat this woman against her will? The moral issues in a case should be central to the deliberation. It is reported that the use of a method for structuring the conversation may be of help here. It also guarantees that all relevant perspectives are heard and that morally relevant aspects are weighed and dealt with [16, 32].

#### A supportive organization

An organization supportive of MCD is a health care organization where MCD is supported and anchored both top-down and bottom-up. Support from upper management is essential [4], but local coordinators should also be convinced of the importance of MCD and coordinate the scheduling, for example, in a ward’s action plan [7, 26, 32]. Deliberations should not be organized on an ad hoc basis only but are preferably integrated into an existing organizational structure [4], for instance, by a scheduling format [27]. Dauwerse et al. [33] emphasized the importance of structurally organizing MCD, as this prevents attention for ethics from being superficial.

### Changes that are brought about on a personal and inter-professional level

Based on our qualitative analysis, we identified several themes and subthemes in this second cluster, which are illustrated in Fig. 2. The changes are related to professional’s feelings, an improved understanding, and the awareness of the moral dimension in one’s work.

### Feelings of professionals

#### Feeling relieved of the burden of moral issues

MCD functions as a forum to speak freely about concerns without being judged and without the primary goal of coming to a concrete result or decision [8, 29]. It can be a relief for participants to *“finally be[ing] able to talk about ethical issues rather than seeing them buried in concerns about clinical care”* [28] (p.559). In addition, doctors reported feeling relieved by being able to share the responsibility for a decision with a multidisciplinary team [15]. Finally, several studies relate participating in an MCD to the reduction of ‘moral distress’ [8, 9, 11, 34]. It was found that participants reported feeling less emotionally distressed or captured by the dilemma [5] and that MCD reduced their moral burden, especially in complex cases [9, 15, 34]. It was also found that participants learned to avoid focusing on solutions [4]. It can be unburdening to talk about dilemmas without having to reach a decision or solution, and to be able to acknowledge the sometimes tragic circumstances in care practice [8]. However, Tanner et al. [15] point out that some professionals might feel an increase in burden due to a lack of mutual agreement, indecisiveness, or having to take multiple perspectives into account.

#### Feeling related to other professionals

As a result of freely sharing experiences and opinions during MCD [17, 32], professionals feel more related to each other [14] and have a more open inter-professional connection [4, 8, 32, 33]. In MCDs, a sense of togetherness is experienced, as participation implies a willingness to both ask and give support [6, 12]. This often is a starting point for trust [7, 12] and not feeling alone in your concerns as a professional [6, 15, 29, 32, 35]. Instead of struggling alone, team members work out a dilemma together [32]. Söderhamn et al. [17] found that participants, as well as outsiders, observed that participants dared to *“speak their minds”* more after MCD. In addition, more informal communication in the wards and at the bedside has been reported [8, 11]. Another illustration of an increased sense of cohesion within a team is that professionals felt freer to address one another more often and earlier with moral issues [7]. In another study, however, participants perceived a gap between themselves and their colleagues who had no experience with MCD, which complicated the dialogue among colleagues [8].

This relatedness is also illustrated in the way in which a team works together in caring for patients after MCD. In patient care, professional action is often accompanied by emotions – for example, doctors’ loneliness in trying to make the right decisions or nurses’ feelings of powerlessness and frustrations [29, 35]. Svantesson et al. [35] found that a group deliberation confirmed participants’ observations concerning how far doctors and nurses stand apart from each other. However, several studies have illustrated a relation between MCD and improved inter-professional collaboration. Group deliberations stimulate awareness of the need for uniformity regarding treatment policy [6]. Different medical professionals adapted and improved their interdisciplinary discussions based on earlier experiences in MCD [15]. Some doctors became aware of the opportunity and their responsibility to explain their motives for continuing life-sustaining treatment in MCD [29]. A more transparent communication about goals and decisions was seen as a possibility to better attend patients due to their improved understanding of the medical situation [15]. Decisions were more easily accepted and carried out [26]. Furthermore, nurses knew how to raise a theme in an interdisciplinary context more effectively than before participating in MCD. Doctors tended to respond to nurses who raised a problem sooner than before participating [15]. It can be a positive and empowering experience for professionals, especially nurses, when voicing one’s opinions is not taboo anymore and instead, one’s perspective is taken seriously and understood by others in the decision making process [6, 7, 15, 26, 32, 36]. Some studies have indicated that MCD leads to thinking more about personal involvement and responsibilities. This includes both setting boundaries to prevent feeling too involved [6], as well as loosening boundaries by not blaming others but sharing responsibility instead [35].

#### Feeling confident

Several studies showed that professionals reported feeling more confident in their work [5, 32, 34], for example, through finding their own approach validated during MCD or through the experience of hearing that others feel the same way about aspects of certain cases [11]. Additionally, understanding all alternatives and weighing them by means of a conversation method or format with specific steps reassures professionals that the decision-making is sound [16] and it gives them *“peace of mind”* [9]. After a deliberation, participants are more inclined to be straightforward and transparent towards colleagues or patients [6]. Seeing alternatives and developing a critical attitude is also associated with confidence to act in future situations [29]. In one study, this resulted in professionals being more assertive and even firm with noncompliant patients [35]. It can be a positive experience for participants and can even be felt as a need or wish to achieve a consensus in the group, especially in difficult cases [30, 35, 36]. Participants in the study of Bernthal et al. [16] reported the deliberation method as being effective for achieving such consensus about how to act.

However, a deeper understanding of a problem made some professionals considerably less certain of the validity of their own approach [28]. When MCD produces more questions than answers, professionals who seek consensus or concrete solutions for problems directly related to their daily practice might become disappointed and frustrated [8, 29, 31]. An MCD will be more successful when participants accept that ‘easy’ answers on how one should act are uncommon and realize that it can still be safe to see different alternatives in a case without reaching a consensus [14]. Deviating from habits or existing policies can be a challenge for participants [9]. Additionally, Van der Dam et al. [8] observed a difference in confidence that was related to a difference in the ability to talk about moral issues in the group. Especially in the first meetings, professionals who were morally more competent felt frustrated and impatient in an MCD with less competent colleagues. Such insecurity can prevent informal communication on moral issues. This example stresses the importance of a safe atmosphere in MCD.

### Understanding by professionals

Speaking and listening to each other in an MCD not only changes feelings, but also has an impact on one’s understanding. We identified three types of understanding by professionals: understanding the perspectives of colleagues, understanding one’s own perspective and understanding the moral issue at stake.

#### Understanding the perspectives of colleagues

Multidisciplinary MCDs are considered a helpful and positive learning experience [13, 32]. In line with the findings about feeling more related with each other, during MCDs, professionals get to better understand one another’s considerations and actions [7, 17, 32]. Professionals become more familiar with each other’s daily work, values, norms and moral struggles [8, 9, 26]. For some participants, it is an eye-opener that colleagues struggle with moral issues as well and in a variety of ways [9, 17]. Furthermore, professionals learn to acknowledge, appreciate and respect the opinions of colleagues and patients to a greater extent [4, 16, 17]. MCD helps them to relate to viewpoints that are not necessarily their own, thus developing a broader perspective on the – sometimes seemingly simple – case at hand [4–8, 11–14]. This will be elaborated further in the paragraph ‘*Understanding the moral issue at stake’*. In addition, by improving professionals’ mutual understanding and understanding of a decision, MCD reduces conflicts [4, 6, 15, 32] and leads to more solidarity, respect, tolerance, collegial support and cooperation [17, 32].

However, one study reported participants struggling to put themselves in someone else’s position [9]. Another study found that a difference in cultural backgrounds was seen as a threat instead of an enriching point of view [32].

#### Understanding one’s own perspective

MCD supports professionals in critically reflecting on and becoming more aware of their own assumptions, intentions, and actions regarding patient cases [4, 7, 14, 17, 35]. This was reported, for instance, with regard to verbal and nonverbal behavior towards (aggressive) patients [17, 35]. According to Van der Dam et al. [9], participants developed *“a more exploratory attitude”* (p. 129). Instead of following old routines and acting on ‘automatic pilot’, professionals are more inclined to question their practices or previous understandings of situations [17, 26, 32, 35]. As a result, nuances can be applied to personal opinions [6].

#### Understanding the moral issue at stake

MCD is not only considered helpful to better understand the perspectives of colleagues and see their struggles with moral issues in general. Several studies have shown that a structured MCD approach helps to clarify and comprehend the specific moral problem at stake [9, 14, 27, 29, 34]. Weighing new information and different arguments – including pros and cons – generally offers a more integrated and holistic view [29, 32]. Instead of working towards ‘the’ right answer or a concrete solution, healthcare professionals learn to see the complexity and multidimensionality in cases [4, 9, 35]. However, two studies showed that MCD did not lead to new insights or questions for participants, or to a lesser extent than was expected [35, 36].

According to some authors, it is this variety of perspectives in the joint deliberation that enhances the moral investigation of the case [8, 9], which is believed to positively influence the quality of care [9]. Van der Dam et al. [8] suggested that reflecting by yourself or with only your own (mono-disciplinary) colleagues lacks this richness of different perspectives. Grönlund et al. [12] observed that through multi-perspective dialogue, new ways of thinking about the specific patient and his or her situation emerged. In general, MCD seems to provide a better understanding of responsibilities and ethical issues in patient care [4, 11, 13, 31, 32, 35]. Some participants develop new ways of thinking about moral problems [28] – especially more systematic and critical approaches [4]. Such an increased understanding can lead to new or better solutions regarding patient cases [7, 32]. However, in several studies, little or no change in opinion about patient cases was reported after an MCD [14–16, 28, 31].

### Awareness of the moral dimension of one’s profession

We identified two types of awareness: awareness of the moral dimension of caring and awareness of the importance of reflection.

#### Awareness of the moral dimension of caring

Participating in MCD results in more attention and more sensitivity to moral issues in general [4, 15, 17, 32]. Participants seemed to think more about reasons, arguments and “gray areas” in their work [4, 14]. Several studies report that group deliberations stimulated creativity in thinking, which resulted in alternative ideas and possibilities [8, 9, 12, 32]. Recognizing and articulating moral issues can be hard for professionals, as it is sometimes assumed that such issues only have to do with ‘difficult patients’ in the wards. However, MCD helps participants to see the variety of moral issues in their professional practice (from everyday problems to managerial questions) and provides insights regarding the moral complexity in seemingly simple or practical cases [4]. For some participants, it became easier over time to write down focused cases [9]. According to the categorization by Dauwerse et al. [33], so-called *“explicit ethics support”,* which includes MCD, places ethical issues and the ethical dimension of care structurally ‘on the agenda’.

Additionally, in multiple studies, MCD is related to the improvement of one’s competence in addressing and managing moral issues [5, 11, 17, 32, 34], for example, by dealing with these issues quickly, more fully and without frustration [12, 13]. Some professionals reported that they felt it became easier to contact their team leader in case of future problems or ideas [7]. As participants learned to join in a moral dialogue, their moral and reasoning skills were trained (e.g., listening, postponing initial judgments, not primarily wanting to convince others, thinking through a dilemma and asking questions) [4, 5]. It seems that ethics education correlates with a greater sense of moral agency, but as Wocial et al. [11] indicated: *“It is not clear (…) whether participation in UBECs [unit-based ethics conversations] leads nurses to act on their moral agency, or if those who are more likely to act on their sense of moral agency are more likely to attend a UBEC.”* (p. 53).

#### Awareness of the importance of reflection

In several studies, participants in MCD stressed the importance of, and the need for, timely and regularly scheduled reflection on their work [5, 7, 11, 17, 35], as opposed to immediately acting in a complex situation [6]. Initially, participants may feel ambiguous about MCD, but participating in deliberation creates an appreciation of MCD [6, 9]. In the study of Söderhamn et al. [17], the combination of both regular meetings and a five minute-method during the day was considered helpful to encourage reflection in everyday practice.

### Changes that are brought about in caring for patients and families

As was elaborated previously with regard to the first cluster of changes in understanding by professionals, MCD may stimulate new ways of thinking about the case at hand. In addition, we identified two ways in which professionals’ caring for patients can be influenced by MCD.

#### Profession-related changes

There are some indications of the impact of MCD on one’s profession. Some studies stated that people can become better healthcare professionals through MCD [17, 26] or that MCD was considered – broadly speaking – helpful for the job or helped participants to gain insight in what is truly important in their work [26, 36]. Additionally, one study showed that after moral reflection, healthcare professionals were more focused on further professionalization, for instance, wanting to learn more about how to provide the best possible patient care [17]. Söderhamn et al. [17] revealed three factors that predict whether or not ethical reflection is valued by professionals: professionals who are older, who have a higher position and who have more experience with such reflections consider MCD to be meaningful in the workplace.

The included studies are ambiguous regarding whether systematic reflection leads to more organizational profession-related changes, such as reduced absenteeism and increased job interest. Lillemoen and Pedersen [32] found that managers and facilitators were confident about this impact, but staff members doubted it. Tanner et al. [15] found that a Swiss ethics program led to decrease in distress in professionals, thereby adding to job satisfaction. This, in turn, decreased frustration and dissatisfaction among nurses.

A change in one’s professional opinion or attitude due to MCD is described by several studies, but in what way this change comes about is less clear [5, 12]. Participants were more critical towards their practice and managers felt more challenged by their employees [32]. Furthermore, experiences of no impact on daily work were reported as well [6, 14].

#### Quality of patient care

The included studies indicate that MCD may influence the quality of patient care. We have divided these results into the impact on the interaction with patients and families and the impact on medical technical care of patients. According to healthcare professionals, through MCD, they developed an enriched understanding of their patients’ situations [9, 32, 35]. Participants reported being more aware of patients’ and families’ rights in a decision-making process [27, 32] and thinking more about their perspectives, wishes, and needs [14, 26, 32]. Meyer-Zehnder et al. [34] indicated an educational effect when MCD takes place regularly, as patient wishes are actually verified and addressed sooner. Participants in another study [17] also reported being more aware of their own verbal and body language, which resulted in more personalized care, more respect and their seeing patients as more than their diagnosis. Some of the staff who did not participate in the ethical reflections in this study observed this change in their colleagues’ behavior as well. Tanner et al. [15] found more support for a mutual and documented decision as a team, for example, towards patients and families. Staff described a relation between MCD and a decreased use of coercion towards their patients [32]. Furthermore, an increased awareness of patients’ wishes led to an openness towards patient and proxy participation, with professionals seeing or hearing patients more [32], and a better representation of parents’ opinions in the decision making process about neonates [27].

In addition to changing the interaction with patients and families, MCD can also influence medical technical care for patients. A better understanding of the patient may lead to more adequate recommendations regarding a patient case [13]. Jehle and Jurchah [13] found that reflection helped with decision making and led to concrete recommendations and actions in a specific situation, thus refining care plans and ensuring they were agreed upon by families, patients, and the team. Additionally, MCD was found to support acting faster and providing better nursing care in similar cases [26]. The study of Baumann-Hölzle et al. [30] showed a concrete change in medical care after MCD: a shortening of futile intensive care compared to a control group. According to Baumann-Hölzle et al., this could be interpreted as limiting suffering in infants destined to die.

### Changes that are brought about on an organizational level

The last theme we identified is professional attention to ethics on an organizational level. In one study, it was found that group deliberations in psychiatric outpatient clinics did not lead to statistically significant changes in the so-called ‘ethical climate’, as measured with a specific survey [18]. However, several studies report an expansion of (informal) discussions and rounds after moral deliberation had taken place [26, 28, 31, 32].

## Discussion

Based on a qualitative analysis of 25 empirical papers, we have gained an overview of what is known about the impact of MCD. The results consist of four clusters of themes we found in the literature (see Fig. 2):

1. Facilitators and barriers in the preparation and context of MCD include the following: a safe and open atmosphere created by a facilitator, a concrete case, commitment of participants, a focus on the moral dimension, and a supportive organization. This is also underpinned by recent research in municipal healthcare, which showed that a systematic and supported approach is helpful in facilitating reflection groups [24]. The facilitator appeared to be the most important facilitating factor.
2. Changes that are brought about on a personal and inter-professional level are concerned with the following: feeling relieved, feeling related to other professionals and feeling confident; understanding the perspectives of colleagues, understanding one’s own perspective and understanding the moral issue at stake; and awareness of the moral dimension and awareness of the importance of reflection. Most of the reported impact is on this inter-professional level. This tells us how healthcare professionals experience participating in an MCD and what they believe is the value of an MCD for dealing with ethical issues, as individuals and as a team. This is in line with what healthcare professionals perceive as important outcomes prior to participating in MCD: ‘more open communication’, ‘better mutual understanding’, ‘concrete actions’, ‘see the situation from different perspectives’, ‘consensus on how to manage the situation’ and ‘find more courses of action’ [21]. Interestingly, despite the daily practice of (multidisciplinary) collaboration in the field of healthcare, this review shows that separate sessions on work-related moral dilemmas are helpful to be able to actually get to know each other’s perspectives and find some relief in that. Apparently, there is a lack of this kind of sharing in daily work. We have to take into account, notwithstanding the mainly positive impact that is reported, that an MCD does not always leads to a decrease in one’s mental or emotional burden. We found that it can be challenging for healthcare professionals to deviate from routines or to see new perspectives. Professionals may feel ambiguous about participating or frustrated when a deliberation does not lead to concrete decisions or consensus. Our findings showed that for some, a deliberation does not result in new insights or changes in opinion, which might add to the ambiguous attitude with regard to participating. The attitude needed for MCD – which requires among other things the willingness to take a step back and explore the moral issue – cannot always be yielded by participants. In our own experience within our hospital, if MCD does not take place in a safe and open atmosphere with committed participants, it is likely to only add to the tension within the team.
3. Changes that are brought about in caring for patients and families are concerned with one’s profession and quality of patient care. Remarkably, this cluster of themes was rather small in comparison with the second cluster, and we found little evidence for a concrete impact of MCD on patient care. In the field of clinical ethics, sometimes rather big claims are made. Karlsen et al. [24] summarized previous research that indicated that staff, managers, and facilitators agree on the relation between ethics reflection groups, a positive impact on work environment, and an increase in quality of care, for example, through the participants’ increased ability to see alternative courses of action and make better decisions [24]. Nevertheless, there is limited empirical evidence with regard to the changes that are *actually* brought about in caring practices after the group conversation has taken place.
4. Lastly, we identified some changes that are brought about on an organizational level. This cluster was equally small. This is in line with the observation of Silén et al. [18] that studies have not yet been able to demonstrate this presumed positive relation between ethics rounds and improvements in the work environment, such as an improved ethical climate, less burnouts or increased job satisfaction. This lack of evidence remains a challenge for the field.

### Not all kinds of impacts can or should be measured

Our overview can help to gain insight into the strengths and weaknesses of MCD, as well as determine blind spots in MCD research. In 1977, Levine et al. [31] stated that the impact of moral deliberation on patient care is difficult to assess. Concrete changes might be hard to grasp with empirical investigation of concepts such as ‘quality of care’. Perhaps, as suggested by Silén et al. [14], the impact on measurable outcomes is mediated by communication and collaboration patterns, which can, in turn, be influenced by moral case deliberation. Thus, one should carefully operationalize ‘improved quality of care’ in further research. In addition, it is debatable whether it is right to justify the practice of MCD in terms of efficiency, quality improvement or other ‘hard’ impacts. We believe the added value of moral case deliberation is ‘soft’ or intangible by nature, and more difficult to pin down in measurable units. Perhaps such a deliberation has a value in and of itself. In that case, it is meaningless to try to measure this ‘soft’ kind of impact in quantified terms or to translate it to specific managerial categories. That might only lead to an (undesired) top-down focus on predefined outcomes, which could diminish the value of MCD. A bottom-up approach is preferable: desired outcomes should not be defined by external stakeholders, but participants themselves should be active in setting the agenda in evaluation studies [20]. De Snoo-Trimp et al. [21], for example, investigated what healthcare professionals themselves perceive as important outcomes.

### The impact is often based on self-reports by participants

The involvement of participants in evaluating MCD is also reflected in the methodologies of the included studies in our review: the found impact is mostly based on self-reports by healthcare professionals in surveys, focus groups, and interviews. However, one should keep in mind that positive evaluations of participants do not necessarily imply that a group deliberation results in concrete changes in the way they treat their patients. In addition, positive evaluations we found might stem from a source of bias, as sometimes the researcher was the coordinator of the implementation and the facilitator of the conversation as well, which might have elicited socially desired behavior. Furthermore, in some papers, the study sample consisted of people who were willing to participate in MCD. This could result in sampling bias, as professionals who participate usually have a positive attitude towards MCD, and their self-reports will reflect this attitude.

Finally, in our review, outcomes with regard to ethics in the organization seem to be the most abstract. This might also be due to the self-reports, since healthcare professionals might not be able to give detailed information about the organization as a whole.

### Implications for further research

If concrete changes are expected with regard to quality of patient care, then one should not only investigate the perspective of professionals but also study the effects as experienced by the patients themselves. Specifically, there is a need for further qualitative research, as we should study the complex care practice which might be changed in a subtle way by MCD. Several authors suggest obtaining a more nuanced picture by using research designs such as a control-group, observational studies [7] or a mixed method design [18]. An example of a recently developed survey is the ‘Euro-MCD’, which investigates participants’ perceived importance of MCD outcomes [37]. We argue that the design of further research should rely heavily on qualitative methods. The positive contribution of qualitative research in the field of clinical ethics support services is further elaborated by Wäscher et al. [38].

Qualitative research should interfere as little as possible in existing practices [39]. This implies strictly separating the role of researcher and facilitator to prevent influencing evaluations from participants. In addition to this, it is important to study the reasons why people waive participation in MCD. With qualitative methods, one can investigate the different perspectives of healthcare professionals, including those who do not want to participate, patients/clients, proxies and others. This might show the actual continuation of MCD in health care practice and in the ‘ethical climate’ of the organization as a whole.

### Implications for practice

Considering the impact of MCD with regard to healthcare professionals feeling more related to one another, a critical thought might arise: “Could regular team meetings not generate similar feelings?” Based on our analysis, we believe that the difference between MCD and other meetings and the added value of MCD lies in its structured approach of freely exploring the moral question at stake, without having to reach a concrete solution or decision. We consider it to be important that all professionals involved in the case or issue join the conversation. In our experience, a structured method and a facilitator are essential elements to create the required open and safe atmosphere and to guarantee a careful critical-ethical analysis from all (multidisciplinary) perspectives. A regular team meeting might result in more cohesion and relatedness but likely in a less thorough way, when compared to a group conversation in which people have a dialogue on a moral issue.

### Strengths and limitations

We adopted a thorough and systematic approach in reviewing the existing literature about the impact of MCD, based on ongoing discussion between the authors. To our knowledge, a literature review of this type has not been conducted before. Our review seems to appeal to a need in practice to account for the value and impact of clinical ethics support. Furthermore, we aim to fill a gap in research with regard to conceptual ambiguity in forms of clinical ethics support services, which is also illustrated by the literature review of Rasoal et al. [2].

However, some limitations should be taken into account when reading this paper. A first limitation is a possible bias in the studies we included. In some papers, the study sample only consisted of people who were willing to participate in MCD. This could results in bias, as professionals who participate are usually favorable towards MCD, and their self-reports are likely to provide a positive outlook. Thus, it is important to investigate which professionals waive participation and for what reason. Secondly, our search was limited by our definition of MCD. Given the conceptual ambiguity in the field of clinical ethics support services, it would be worth-wile to make an inventory of all the sorts of deliberations used in practice (independently of empirical research), for example through a questionnaire at international symposia or by means of a global Delphi round within our ethics networks. A third limitation is the absence of an evaluation of the methodological soundness of the included papers. This should be kept in mind when reading and interpreting our results.

## Conclusions

With this literature review, we aimed to present an overview of the empirical evidence for both the positive and negative impacts of MCD. It was shown that MCD brings about changes in practice, mostly for the professional in inter-professional interactions with regard to one’s feelings of relief, relatedness and confidence; understanding of the perspectives of colleagues, one’s own perspective and the moral issue at stake; and awareness of the moral dimension of one’s work and awareness of the importance of reflection. Most reported changes were considered positive, although challenges, frustrations and absence of change were also reported. Empirical evidence of a concrete impact on the quality of patient care is limited and is mostly based on self-reports. With patient-focused and methodologically sound qualitative research, the practice and the value of MCD in healthcare settings can be better understood, thus making a stronger case for this kind of ethics support.

## Abbreviations

CES: Clinical ethics support
MCD: Moral case deliberation
